# Neonatal morbidity and mortality in Hargeisa, Somaliland: an observational, hospital based study

**DOI:** 10.11604/pamj.2020.37.3.24741

**Published:** 2020-09-02

**Authors:** Karen Marie Lundeby, Espen Heen, Mohammed Mosa, Abdirashid Abdi, Ketil Størdal

**Affiliations:** 1 Hargeisa Group Hospital, Hargeisa, Somaliland,; 2 Oslo University Hospital, Oslo, Norway,; 3 University of Oslo, Oslo, Norway,; 4 Ohio State University Wexner Medical Center, Columbus, USA,; 5 Norwegian Institute of Public Health, Oslo, Norway,; 6 Ostfold Hospital Trust, Sarpsborg, Norway

**Keywords:** Neonatal mortality, prematurity, low birth weight, exclusive breastfeeding, neonatal unit, Somaliland

## Abstract

**Introduction:**

Hargeisa Group Hospital, Somaliland, opened a neonatal unit in 2013. We aimed to study causes of admission, risk factors for neonatal death and post-discharge care to address modifiable factors.

**Methods:**

we analysed hospital records from June-October 2013 (n=164). In addition, we reached primary caregivers of 94 patients for further information after discharge.

**Results:**

of the 164 patients, 65% were male, 31% weighed <2500 grams, 16% were premature, 43% were exposed to meconium and 29% had premature rupture of membranes (PROM). Twenty-seven percent were admitted after caesarean section and 36% had been bag-mask ventilated. The most common diagnoses for admission were asphyxia (34%), respiratory distress (27%), sepsis (16%) and prematurity (15%). The mortality before discharge was 15%, 23/1430 (1.6%) of live-born at the hospital. Half of the admitted preterm babies died (RR for death for preterm vs term born 4.6, 95% CI 2.3-9.0) as well as 28% of the patients with birth weight <2500 grams (RR 2.1, 95% CI 1.0-4.2). The mortality rate with or without PROM was 29% vs 15% (RR 2.0, 95% CI 1.0-3.9). At 28 days of age, 34% of the patients were exclusively breastfed and 44% had not yet been vaccinated. Diarrhoea, vomiting and/or respiratory distress after discharge were reported for 44%.

**Conclusion:**

prematurity and low birth weight were important risk factors for neonatal death in this cohort, contributing to the high mortality rate. Low numbers of exclusively breastfed and vaccinated infants are also issues of concern to be targeted in the peri- and postnatal care.

## Introduction

Deaths within the first month of life are estimated to represent 46% of under-five deaths globally. The percentage is increasing due to a slower decline than seen in post-neonatal deaths [1]. Every country is therefore challenged by the new UN Sustainable Development Goals to bring the neonatal mortality rate (NMR) down to 12 per 1000 live births by 2030 [2]. The region of Somaliland is estimated to have a NMR of 42 per 1000 live births as reported by the United Nations Children's Fund's Multiple Indicator Cluster Survey (MICS) of 2011 [3]. Estimates from 2017 indicate slightly better NMR at 39 per 1000 live births for the whole of Somalia [1]. Hargeisa Group Hospital (HGH), Somaliland's only governmental tertiary hospital, was built in 1953. Today it has a capacity of about 450 beds, including medical and surgical wards and serves about 5500 deliveries a year [4]. According to the hospital routine registration for the year 2013 the stillbirth rate was 7% [5]. Data separating fresh and macerated stillbirths were not available. Fifteen percent of all deliveries were caesarean sections. Data on neonatal health from birth, based on hospital studies, have, to the best of our knowledge, not been available from Somaliland. We therefore aimed to describe neonatal morbidity and mortality, to study risk factors for neonatal death as well as to assess post-discharge care, based on information from patients admitted to the neonatal unit at Hargeisa Group Hospital.

## Methods

**Figure 1:**
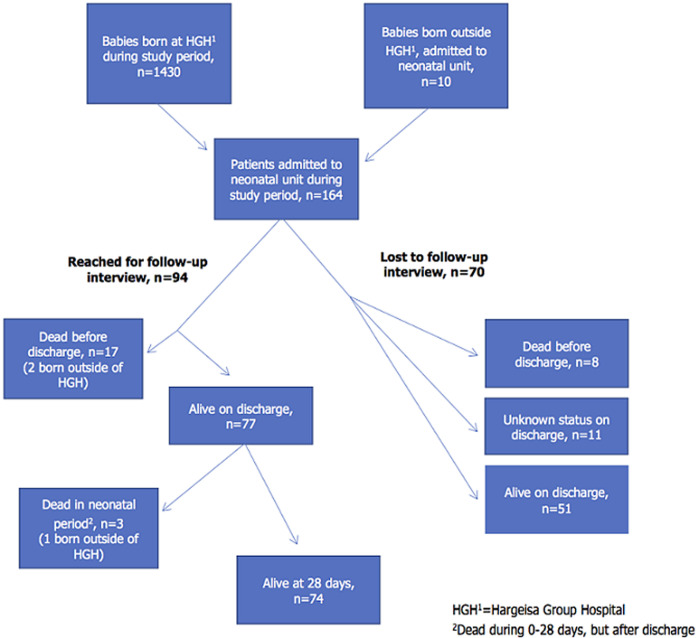
flow diagram of neonatal study recruitment and adjacent mortality numbers at Hargeisa Group Hospital, Somaliland, June-October 2013

**Setting and study design:** up until early 2013 HGH did not have any dedicated place or staff to take care of sick new-borns. It was therefore decided to establish a small neonatal unit. Local nurses and doctors were, through a three months intensive program, trained in how to diagnose and treat different neonatal problems using established WHO guidelines, with “Managing newborn problems: a guide for doctors, nurses and midwives” as the key manual [6]. During the study period presented in this article, there was no access to mobile X-ray or microbiological culture from blood or spinal fluid and the laboratory service was open only during daytime. Most diagnoses were based on history and clinical judgement, with minimal supplemental investigations. The neonatal unit was established inside the maternity ward, making it possible for stable babies to stay with their mothers. This observational study is based on hospital records from 164 patients admitted to the neonatal unit from June 1 to October 31, 2013, as well as a structured interview after discharge. The records show that 1430 babies were born in the hospital during the same period. Table 1 provides the contextual data of the hospital maternity service for the study period reported in this article [5]. All admitted patients were registered with sex, birth weight, estimated gestational age, mode of delivery, interventions immediately after birth, tentative diagnoses and outcome. We also contacted the primary caregivers when the baby would be at least 28 days old. A telephonic interview was done with a structured questionnaire translated from English into Somali by two bilingual medical doctors. This questionnaire gave us information about the child´s family background, breast feeding, vaccination status as well as morbidity and/or mortality after discharge. The interviews were done in Somali by local medical students. They were trained with mock interviews to enhance confidence and accuracy before the caregivers were contacted. We managed to reach 90 primary caregivers of 94 neonates. Most of the caregivers were the biological mothers of the patients. Figure 1 describes the recruitment and flow of the participants.

**Table 1:** routine data from newborns at the maternity ward at Hargeisa Group Hospital, Somaliland 2013

Maternity services	Annual, N (%)	June-October, N (%)
Referral from MCH^1^	330 (10)	125 (9)
Noninstrumental vaginal deliveries	2703 (84)	1183 (83)
Instrumental vaginal deliveries	48 (1.5)	31 (2)
Caesarean Sections	478 (15)	216 (15)
Total^2^	3229	1430
**Outcomes^3^**		
Live births	2995 (93)	1323 (92.5)
Stillbirths	235 (7)	101 (7)
Newborn (early neonatal) deaths	48 (1.5)	23 (1.6)
Registered sick babies after birth	369 (11.5)	183 (13)
Babies below 2500g weight	196 (6)	94 (6.6)

1 MCH=Maternal Child Health clinics; ^2^Total number of deliveries in this table do not add up from live births+stillbirths, but are reported here as recorded in the hospital records; ^3^The verified perinatal mortality from June-October 2013 (including confirmed deaths post discharge) was 8.7%

**Definitions and diagnostic tools:** a neonatal death was recorded if a baby, perceived to be alive at birth, died within 28 days of age. A stillbirth was a baby born with no signs of life at or after 28 weeks' gestation, in line with the WHO definition [7]. We used birth weight of less than 2500 grams to define low birth weight and estimated gestational age (GA) less than 37 weeks as prematurity. The gestational age was estimated using Ballard score [8], as well as term date, if known. Rupture of membranes of more than 18 hours before birth was defined as premature rupture of membranes (PROM). Apgar scores were not used systematically and have therefore not been included in our analyses. The diagnoses were based on the birth history, the status of the newborn, any signs of infection and other relevant clinical findings. Self-discharge was recorded if the patient left the hospital against medical advice or without the doctors´ knowledge.

**Statistical analysis:** the data was plotted in EpiData v.4.0. The 94 patients with extended data from the questionnaire were plotted twice to ensure correctness. Further descriptive analyses were done with Microsoft Excel v.15.32 and IBM SPSS Statistics 25. Interval data were grouped based on relevant clinical categories. Nominal and categorical data were analysed using Pearson Chi-square or Fishers exact test for independence. Post-hoc analysis was done on significant findings. Binary regression analyses with robust cluster correction for twin births were done with Stata version 14 (StataCorp LP, college station, Texas, USA). We investigated the possibility of systematic differences between the complete and incomplete cases. Sensitivity analyses and stratified sub-group analyses were done to assess external validity in mortality data. A p-value of less than 0.05 was regarded as significant.

**Ethical approval:** the Somaliland Minister of Health and the hospital director of HGH gave the ethical approval for this study in 2013. All the caregivers contacted after discharge had to give their informed consent for an interview before any further questions were asked. The patients were de-identified before statistical analyses. All work was conducted based on the declaration of Helsinki ethical principles for research.

## Results

**Admission data:** of the 164 patients admitted to the neonatal unit, 65% were male. Ninety-four percent were born at HGH, the remaining ten patients came from local clinics or were born at home (Table 2). Eight of these ten patients were admitted within two days of birth. The rate of self-discharge was 29%. The mean birth weight was 2694g (range: 800-6000g) and 31% weighed less than 2500g. Sixteen percent were born premature (GA<37 weeks). As Table 2shows, 27% were born by caesarean section, 29% had PROM and 36% were ventilated with bag-mask before admission. Comparing the subgroup reached for follow-up after 28 days post-partum (n=94) and the sub-group not reached (n=70), the latter had a higher proportion of girls with less risk factors for postnatal disease than those reached for interview. Pearson Chi-square tests showed significant differences between the two groups for sex (p=0.01; OR 2.74) and premature rupture of membranes (p=0.01; OR 2.75) (Table 2). A higher percentage of the patients not reached were bagged compared to those reached for follow-up. Table 3 shows the clinical diagnoses on admission. Asphyxia was the most frequent, affecting 34% of the neonates. Respiratory problems, clinical sepsis and prematurity were all common diagnoses.

**Table 2:** admission characteristics of 164 patients at the neonatal unit at Hargeisa Group Hospital, Somaliland, June-October 2013

Categories	All within the category, N (%)^2^	Follow-up questionnaire		P-value for differences between reached and not reached (Chi-squared^3^ or Fisher^4^)
		94 reached, N (%)^2^	70 not reached, N (%)^2^	
**Male^1^**	**101 (65)**	**66 (75)**	**35 (52)**	**0.01^3^**
**Birth weight^1^**				**0.56^3^**
<1500g	21 (14)	13 (15)	8 (12)	
1500-2499g	26 (17)	15 (17)	11 (17)	
2500-3499g	78 (52)	41 (48)	37 (59)	
≥3500g	25 (17)	17 (20)	8 (12)	
**Estimated gestational age^1^**				**0.41^4^**
<32	7 (5)	5 (6)	2 (3)	
32-36+6	17 (11)	12 (14)	5 (8)	
≥37	130 (84)	71 (80)	59 (89)	
**Place of birth^1^**				**0.74^4^**
Born at home or local health clinic	10 (6)	5 (5)	5 (7)	
**Meconium exposure^1^**	**66 (43)**	**39 (43)**	**27 (44)**	**0.86^3^**
**Premature rupture of membranes^1^**	**43 (29)**	**32 (37)**	**11 (17)**	**0.01^3^**
**Mode of delivery^1^**				**0.11^4^**
Normal vaginal delivery	102 (63)	54 (59)	48 (71)	
Instrumental delivery	9 (6)	6 (6)	3 (4)	
Caesarean section	44 (27)	27 (29)	17 (25)	
Other	6 (4)	6 (6)	0 (0)	
**Bag/mask ventilation^1^**	**51 (36)**	**26 (31)**	**25 (42)**	**0.20^3^**

1 :number of patients with missing information within category: sex=9, birth weight=14, estimated gestational age=10, place of birth=3, meconium=12, premature rupture of membranes=14, mode of delivery=3, bag/mask ventilation=21; ^2^:may not add up to 100% due to rounding numbers. Denominator=164; ^3^:calculated with Chi squared test; ^4^:calculated with Fisher's test

**Table 3:** presumptive diagnoses on admission for sick newborns at Hargeisa Group Hospital, Somaliland, June-October 2013

Diagnoses^1^	Number (%) of patients with specific diagnosis, n=164 patients
Asphyxia	56 (34.1)
Respiratory distress (not specified)	45 (27.4)
Clinical sepsis	26 (15.9)
Prematurity	25 (15.2)
Meconium aspiration syndrome	10 (6.1)
Hypoglycaemia	10 (6.1)
Seizures	5 (3.0)
Small for gestational age/feeding problem	4 (2.4)
Bleeding	3 (1.8)
Other^2^	50 (30.5)
Unknown	6 (3.7)

1 Some patients received two different diagnoses. Total number of diagnoses including unknown: n=240; ^2^ Other: hypothermia, icterus, HIV-exposed, low oxygen saturation, anemia, skin rash, head swelling

**Mortality and risk factors for death:**twenty-five patients (15%) died before discharge (two of these were born outside HGH), giving a mortality rate of 16 per 1000 live births in the hospital. Twenty-one died within the first 48 hours of life. In the subgroup with known outcome after one month, three more patients died within the neonatal period (Figure 1). This gave a verified neonatal mortality of 17% among admissions in the neonatal unit. We found that half of the patients admitted to the neonatal ward with GA<37 weeks died. Table 4 shows that the relative risk (RR) for death among preterm infants compared to term born was 4.6 (95% CI 2.3-9.0, p<0.001). A subgroup analysis only including the 94 complete cases, did not change the main conclusion (data not shown). The RR for death associated with prematurity, birth weight and PROM were reduced slightly to 3.3, 1.9 and 1.5, respectively. The patients not reached for follow-up interview (43%) constituted significant (p=0.03) higher proportions in the sub-groups that were self-discharged (60%) or had missing information about type of discharge (47%). Among the self-discharged with complete data (n=19) there were no deaths within 28 days. For the others with incomplete data, the RR for predictive factors of mortality, shown in Table 4, did not differ systematically from the complete cases.

**Table 4:** risk factors and relative risk of neonatal death at Hargeisa Group Hospital, Somaliland, June-October 2013

Neonatal death (n=152)			
Risk factors	Yes^1^, n=28 (%)	No^1^, n=124 (%)	Relative risk (95% confidence interval)^2^
**Sex^1^**			
Male	15 (16)	80 (84)	0.7 (0.3-1.4), p=0.29
Female	11 (23)	37 (77)	Ref.
**Birth weight^1^**			
<2500g	12 (28)	31 (72)	2.1 (1.0-4.2), p=0.05
≥2500g	13 (14)	83 (86)	Ref.
**Prematurity (GA<37)^1^**			
Yes	12 (50)	12 (50)	4.6 (2.3-9.0) p<0.0001
No	13 (11)	106 (89)	Ref.
**Premature rupture of membranes^1^**			
Yes	12 (29)	29 (71)	2.0 (1.0-3.9), p=0.06
No	15 (15)	85 (85)	Ref.
**Meconium exposure^1^**			
Yes	9 (14)	54 (86)	0.6 (0.3-1.3), p=0.23
No	18 (23)	62 (77)	Ref.
**Bag/mask ventilation^1^**			
Yes	11 (22)	39 (78)	1.4 (0.6-2.9), p=0.41
No	14 (16)	73 (84)	Ref.

1 Number of patients with missing information within each category: sex=9, birth weight=13, prematurity=9, premature rupture of membranes=11, meconium=9, bag/mask ventilation=15; ^2^ Analysis with robust cluster correction for twin births. P-value <0.05 regarded as significant

**Family background and possible risk factors after discharge:** among the primary caregivers of the 94 neonates reached for a telephonic interview (four mothers with twins and three relatives that were not the mother), thirteen percent were nomads. Most primary caregivers (57%) were less than 30 years old. Half of them (52%) had never attended any governmental or religious school. More than one-third (34%) of the mothers had experienced death of another child. About half (47%) of the mothers reported they had been given advice about breastfeeding during admission. Sixteen percent were exclusively breastfeeding on discharge, increasing to 34% after 28 days. Ten mothers (14%) were able to exclusively breastfeed their baby throughout the first month of life. In addition to breast milk and formula milk, some reported giving salt, sugar water, honey, goat milk or subaag (local food). Almost half of the primary caregivers reported sickness of the baby between discharge and until one month of age (44%). Of these, diarrhoea, respiratory distress and vomiting were fairly equally distributed. None reported having taken the baby to a traditional or religious healer, but four patients had been given traditional medicine. One out of ten patients had been taken to a medical facility and given treatment within the neonatal period after discharge. Almost half (48%) had received at least one oral polio vaccine (OPV) during the neonatal period and 24% had received BCG (Bacille Calmette-Guerin) vaccine. Forty-four percent had not been given any vaccines within the first month of life. We found no significant associations regarding neonatal mortality and characteristics of the primary caregivers, as shown in Table 5.

**Table 5:** information about primary caregivers and the status of the neonate after discharge for those reached for interview, Somaliland 2013 (n=94)

Characteristics	Alive at 28 days of age, n=74 (%)	Dead within neonatal period, n=20 (%)	Relative risk (95% confidence interval)
**Nomadic lifestyle^1^**			
Primary caregiver living as a nomad	6 (10)	4 (25)	2.2 (0.19-1.09)
Not living as a nomad	54 (90)	12 (75)	1 (ref.)
**Age of primary caregiver in years**^1^			
<20	7 (10)	0 (0)	1 (ref.)
20-29	38 (53)	9 (45)	0.96 (0.1-6.3)
30-39	22 (31)	10 (50)	1.6 (0.2-9.9)
≥40	4 (6)	1 (5)	NA^3^
**Level of education^1^**			
Primary caregiver without any education	38 (52)	11 (55)	1(ref.)
Primary caregiver with some education	35 (48)	9 (45)	0.9 (0.4-2.1)
**Exclusive breastfeeding^1^**			
Exclusive breastfeeding on discharge	14 (19)	1 (50)	NA^3^
Not exclusive breastfeeding on discharge	59(81)	1 (50)	NA^3^
Exclusive breastfeeding at 1 month^2^	25 (34)	NA	NA
Not exclusive breastfeeding at 1 month^2^	49 (66)	NA	NA
**Death of a previous child^1^**			
Mother lost a previous child	24 (34)	8 (44)	1.4 (0.6-3.2)
Mother never lost a previous child or primipara	46 (66)	10 (56)	1(ref.)

^1^ Number of patients with missing information within each category: nomad=17, age=2, education=1, breast feeding on discharge=2, breast feeding at 1 month= 2, death of a previous child=6; ^2^ Those who died before 28 days, n=20, are excluded from analysis; ^3^ Not applicable due to small numbers

## Discussion

In this first study of its kind from Somaliland, we found that prematurity together with birth asphyxia were the most frequent causes for admission. Prematurity and low birth weight were both significant risk factors for neonatal mortality among our patients. The neonatal unit had a mortality of 15% before discharge and the mortality increased to (at least) 17% before the patients reached 28 days of life. Very few mothers were exclusively breastfeeding the first month postpartum and we detected large gaps in vaccine coverage.

**Strengths and weaknesses of the study:** a strength of this study is that the majority of the sick neonates have been followed up after discharge to a minimum of 28 days of life, while most hospital based neonatal mortality studies stop when children are discharged from the ward. The lack of microbiological and available radiological support influenced the accuracy of the diagnoses given. Still, WHO guidelines were actively used to diagnose as correctly as possible in this setting. Even in more resourced settings doctors will conclude on a presumptive admission diagnosis based on the clinical picture, as for sepsis [9]. Ballard score, though never completely accurate, were used to estimate the gestational age [8]. If the term date was known, this was (probably) also used, with limited accuracy. Self-discharge was a big challenge in this setting where the doctors were not available 24/7. The primary caregivers left the hospital with their babies earlier than advised for many different reasons (e.g. needing to take care of other children at home, dependence on getting a lift back to the village). Almost one third of our patients left the hospital before the treatment was finished, making it difficult to evaluate whether the diagnoses fitted the clinical picture and the expected response to treatment. It also caused some missing data about whether the patient was alive or dead at discharge. Of the 70 (43%) that were lost to follow-up, some were out of reach because of a nomadic lifestyle with phone number belonging to a relative in town. This reflects the difficulties of following up patients after discharge for any reason in this context.

Nevertheless, we have put in considerable effort to assess the validity of the follow-up data by showing that no sub-group seems to be worse off regarding registered risk factors for morbidity and mortality (Table 2, sensitivity analysis and stratified risk analysis for mortality). Still, we cannot fully exclude that the 94 complete cases were a positively selected group of the two. The primary caregivers were contacted in November 2013, which was between one and six months after the patients were discharged. Thus, the risk of recall bias was minimized. There is always a possibility that some of the primary caregivers under-reported illness when asked about morbidity after discharge. Some might have feared they would be judged if they reported a sick baby and therefore chose not to report it. On the other hand, they might also, for instance, have over-reported a normal regurgitation as vomiting.

**Comparison with other studies:** to our knowledge, there is a paucity in published research of hospital based neonatal data and outcomes from Somaliland. The closest comparisons are to be found from countries with similar settings [10-13] and global estimates [14,15]. We found a mortality rate of 15% among our patients before discharge. This is comparable to published data from other neonatal units in areas with limited resources as reported by Mèdecins Sans Frontières (MSF) and Dörnemann *et al*. [11]. They found a mortality rate of 17% when looking at eight of their specialised neonatal care units. A hospital based cohort study in the Somali region of Ethiopia, reports of a neonatal mortality rate of only 5.7% [12]. But Zuniga *et al*. found a neonatal mortality rate of 15% among admitted patients to a neonatal unit in Burundi [16] and Aluvaala *et al*. reported an overall mortality rate of 19% in five different Kenyan neonatal units [17]. Adding the three verified deaths after discharge increases the neonatal mortality to 17% among the admitted patients at HGH. We also expect that some of the still-births are misclassified early neonatal deaths with weak signs of life. The overall NMR in this newborn cohort of 1323 babies (Table 1), is therefore probably higher than the crude 16 per 1000 live births. UNICEF´s Multiple Indicator Cluster Survey (MICS) of 2011 estimates the NMR in Somaliland to be 42 per 1000 live births [3]. This survey, however, is based on household interviews and not hospital records.

According to MICS, 67% of mothers in Somaliland give birth at home, thereby indicating that the hospital born cohort is a selected group. Some of the referred women will be high risk labours, probably also contributing to the high prevalence of stillbirths at the hospital. Others, however, will be normal deliveries were the mothers want a safer birth than can be provided at home. Thus, selection of both high and low risk labours will influence the risk for neonatal mortality compared to the national cohort. The additional three patients who died after discharge, within 28 days of age, were less than we expected and this small number, in our opinion, even when adjusting for expected deaths among the patients not reached for follow-up, gives credit to the national staff trying to treat very sick neonates in a resource constrained context.

As expected from other studies, we found the biggest risk factor regarding neonatal mortality to be prematurity [12,18,19]. In a neonatal facility at a referral centre in Uganda, Hedstrom *et al*. found that mortality was inversely proportional with both gestational age and birth weight [20]. We found a lower mortality (17% vs 22%), but birth weight <2500g was also a significant factor for increased risk of death in our study. It should, however, also be mentioned that the neonatal unit in Hargeisa discharged three live patients with a birth weight less than 1000g. One of these patients was still alive after 28 days, the other two were not reached for follow-up. This illustrates, however, that simple, but good basic care of newborn babies can have a great impact even for the most vulnerable groups. PROM was a significant risk factor for neonatal death at HGH, as found at the Moi Teaching and Referral Hospital in Kenya [21]. This was most likely due to an increased risk of infection [22]. We lack data regarding whom of the mothers received antibiotic treatment before delivery. Meconium-stained amniotic fluid was not shown to be a significant risk factor in our study. Looking at the main challenges of our sick neonates, the six diagnoses making up more than 70% of all admission diagnoses, are all in some way or another related to preventable challenges pre- and intrapartum. Prenatal steroids, close monitoring and antibiotic treatment are possible preventive measures that could be addressed.

Many studies have shown how important breast milk is for growth and a healthy immune system in the baby [23], supporting the WHO/UNICEF recommendations of exclusive breastfeeding in the first six months [24]. Nurses and midwives at HGH, both at the neonatal unit and maternity ward, were instructed to promote exclusive breastfeeding. We found that less than half of the mothers reported receiving information about this during their hospital stay. Some mothers might have been too sick to remember the information given, but exclusive breastfeeding is obviously a topic health personnel on all levels need to be better at communicating. The prevalence of exclusive breastfeeding in this study are in line with the MICS Somaliland study [3], where only 13% of children age 0-6 months were exclusively breastfed. The reasons are probably manifold: some premature babies were given formula milk on a nasogastric tube until improvement and the mothers might have continued bottle feeding after discharge. Some of the mothers were also too sick to attend their babies in the neonatal unit and therefore were not able to establish proper breastfeeding before discharge. Cultural beliefs and habits also play their part.

Although vaccination within the neonatal period might not influence mortality during the first month of life, it plays a crucial role in reducing child mortality at large [25]. The WHO vaccination program for Somaliland recommends both oral polio and BCG at birth [26]. Provision of vaccines was lacking at HGH during the study period and only about 2/3 of the neonates in our selected cohort received a minimum of one vaccine at local medical clinics. MICS Somaliland also report of low vaccination coverage in urban areas [3]. Oral polio coverage of 22% (at birth) is less than half our findings; BCG coverage of 40% in the MICS report also includes catch-up vaccinations within the first year of life. The difference in coverage of BCG might also be explained by the reluctance of giving a live vaccine to sick neonates.

The primary caregivers reported that almost half of the 94 patients had been sick after discharge and within their neonatal period. We could, however, not find any significant association between the symptoms registered and the assumed risk factor of lack of breastfeeding. Less surprising was the missing association between morbidity and vaccines, since the observation time was just 28 days.

**Unanswered questions and future research:** this study focused on the sick neonates admitted to the neonatal unit at Hargeisa Group Hospital in 2013. Since then, the Ministry of Health in Somaliland has done a substantial effort to put mother and child health on the agenda. Local health clinics (MCH) have been built and numerous midwives, nurses and doctors have been trained. The neonatal unit at Hargeisa Group Hospital has expanded both regarding facilities and staff due to higher demands. There are considerable efforts in place to further improve the neonatal service in this hospital. A similar study today might therefore find a lower mortality rate than presented here. This study, however, reveals a knowledge gap in peri- and postnatal hospital care in Somaliland and highlights the need for studies on general hospital neonatal cohorts as well as population based studies. There is also a need of follow-up studies of the long-term consequences of neonatal disease and treatment in this context, including neuromotor- and cognitive development. Reasons for self-discharge have not been assessed, but may reflect financial constraints and social consequences of staying away from the family. How to increase coverage of both exclusive breastfeeding and vaccination in this context, should also be addressed in further studies.

## Conclusion

This study, based on admitted patients to a neonatal unit in Somaliland in 2013, confirms that prematurity and low birth weight are important risk factors for neonatal mortality. It also shows that a worrying number of mothers did not exclusively breastfeed their neonates, even though this has been recommended for decades. These are all possible targets for better peri- and postnatal care that might reduce neonatal mortality in this setting. In addition, we found low vaccination coverage, which is another concern for the neonates who survive their first month of life.

### What is known about this topic

- Somaliland and Somalia has one of the highest neonatal mortality rates in the world;
- Prematurity and low birth weight are strong risk factors for neonatal mortality globally;
- With modest interventions, many sick newborns can survive the critical first week of life, even in low income countries.

### What this study adds

- This is the first analysis of 28-day mortality rate for sick neonates treated in a hospital neonatal unit in Somaliland;
- Few mothers of previous sick neonates were exclusively breastfeeding their babies;
- Numbers of neonates dying at home after hospital treatment for peripartum morbidity are small compared to those dying during labour and the first days of life in the hospital.
